# First isolation, identification, phenotypic and genotypic characterization of *Brucella abortus* biovar 3 from dairy cattle in Tanzania

**DOI:** 10.1186/s12917-015-0476-8

**Published:** 2015-07-21

**Authors:** C. Mathew, M. Stokstad, T. B. Johansen, S. Klevar, R. H. Mdegela, G. Mwamengele, P. Michel, L. Escobar, D. Fretin, J. Godfroid

**Affiliations:** Department of Production Animals Clinical Sciences, Norwegian University of Life Science, Oslo, Norway; National Veterinary Institute, Oslo, Norway; Sokoine University of Agriculture, Morogoro, Tanzania; Veterinary and Agrochemical Research Center, Brussels, Belgium; Department of Arctic and Marine Biology, University of Tromsø - the Arctic University of Norway, Faculty of Biosciences, Fisheries and Economics, Research Group of Arctic Infection Biology, Langnes, Postbox 6050, 9037 Tromsø, Norway

**Keywords:** Abortion, Bovine, Biotyping, *Brucella*, Dog, Genotyping, MLVA, Prevalence, Small ruminants, Zoonosis

## Abstract

**Background:**

Brucellosis is a disease of worldwide public health and economic importance. Successful control is based on knowledge of epidemiology and strains present in an area. In developing countries, most investigations are based on serological assays. This study aimed at investigating a dairy herd experiencing abortions in order to establish within-herd seroprevalence to *Brucella* spp., identify, characterize *Brucella* strains by Multiple Loci Variable Number of Tandem Repeats Analysis (MLVA-VNTR) and investigate possible spillover to other species.

**Results:**

The within-herd seroprevalence in cattle (*n* = 200) was 48 % (95 % CI 41–55), using an indirect ELISA, while the Rose Bengal Test (RBT) yielded lower prevalence (21.5 %; 95 % CI 16–27). Two sheep (*n* = 35) and one goat (*n* = 50) were seropositive using ELISA while none of the dogs (*n* = 6) was positive with the RBT. Three *Brucella* were isolated from an aborted fetus and associated membranes. Real time PCR (IS*711*), Bruce-ladder and classical biotyping classified the isolates as *B. abortus* biovar 3. MLVA-VNTR revealed two different but closely related genotypes. The isolates showed unique profiles, providing the first genotypic data from Tanzania. These genotypes were not related to *B. abortus* biovar 3 reference strain Tulya originally isolated from a human patient in Uganda in 1958, unlike the genotypes isolated and characterized recently in Kenya. High within-herd prevalence, isolation of the pathogen and abortion confirm that *B. abortus* is circulating in this herd with cattle as reservoir hosts. A low seroprevalence in sheep and goats suggests a spillover of *B. abortus* from cattle to small ruminants in the herd.

**Conclusions:**

This is the first isolation and characterization of *B. abortus* biovar 3 from a dairy cow with abortion in Tanzania. The origin of the Tanzanian genotypes remain elusive, although they seem to be related to genotypes found in Europe, Turkey and China but not related to *B. abortus* biovar 3 reference strain or genotypes from Kenya. Importantly, replacement heifers are commonly sourced from large farms like this to smallholder farmers, which poses risk of spread of bacteria to other herds. *B. abortus* is a significant zoonotic risk and animal health problem in this production system, therefore further studies on humans is recommended.

## Background

Brucellosis is a zoonotic disease of high economic and public health importance worldwide [1–3]. It is caused by *Brucella* spp. and manifests itself as abortion and infertility in domestic and wild animal species and reduced milk production in cattle. In cattle the disease is mainly caused by *B. abortus.* However, other species of *Brucella* can also be isolated [4–8]. Brucellosis in humans is almost always associated with infected domestic and wild animals or their products and poses more risk to farmers, animal handlers, abattoir workers and veterinarians [9]. It causes a debilitating disease with unspecific symptoms comparable to other febrile conditions such as malaria, which may be chronically disabling. Treatment of human brucellosis is long and costly.

*Brucella* are small (0.5 to 0.7 by 0.6 to 1.5 μm), gram negative, non-motile, non- encapsulated, non-spore forming, rod shaped (coccobacilli) bacteria which are facultative intracellular parasites. The genus shows little variation genetically. To date there are 11 recognized species of *Brucella* which are genetically very similar although each has different host preferences [6]. Six are regarded as classical *Brucella* spp. Four members have recently been classified as additional species [10, 11] and recently the eleventh *Brucella* spp. has been described [12]. Three species are of great zoonotic and economic importance; these are *B. abortus*, *B. melitensis*, and *B. suis* which preferentially infect cattle, small ruminants and swine respectively. Some *Brucella* spp. are further divided into several biovars. So far, *B. abortus* has been subdivided into biovars 1, 2, 3, 4, 5, 6, 7 and 9 [13]. Several biovars of *B. melitensis* (biovar 1, 2, 3) and *B. suis* (biovar 1, 2, 3, 4, 5) are also recognized [14]. *Brucella abortus* biovar 1 accounts for more than 80 % of the total number of isolates worldwide whereas in Africa *B. abortus* biovar 3 has been reported in most of the few published studies [2, 4].

Screening of brucellosis can be performed by serological methods detecting antibodies directed against epitopes associated with the smooth lipopolysaccharide (S-LPS) [5]. Confirmation of the infection is done by culture and isolation of the bacteria. However, this bacterium is difficult to grow and the procedure is time consuming. Furthermore, the procedure poses a risk to laboratory personnel and should be performed in biosafety level 3 laboratories. Nevertheless this method remains the “Gold standard” for diagnosis of brucellosis and *Brucella* infections. Biotyping of *Brucella* spp. provides additional information. Polymerase Chain Reaction (PCR) and other molecular techniques have been developed and have found diagnostic application [1]. Detection of *Brucella* spp. or its DNA provide the only certain diagnosis [5]. Genotyping of *Brucella* spp. can be achieved by Multiple Loci Variable Number of Tandem Repeats Analysis (MLVA-VNT) which shows a very good discriminatory power [14]. Such data can provide molecular epidemiological information for elucidating transmission pattern.

Brucellosis is widely spread in African countries [2, 3, 15, 16]. Serological studies done in different parts of Tanzania indicate that the infection is widely spread in domestic animals, wildlife and human beings [17, 18]. In Tanzania the problem is bigger in pastoral systems and wildlife than in the dairy farming system [18]. Data on isolation of *Brucella* spp. both in humans and animals, with further characterization is scarce. Isolation of *B. abortus* and *B. melitensis* from cattle and small ruminants in Tanzania was reported more than 50 years ago. However characterization of the isolates was not performed [19]. *Brucella melitensis* and *B. abortus* have been isolated and characterized from cattle in Uganda and Kenya. In Tanzania, similar studies need to be performed to trace back the reservoir host species [7, 20]. It is not known whether cattle in Tanzania are infected with *B. abortus* or *B. melitensis* or both. Successful control of brucellosis requires knowledge of its epidemiology in different animal species and the circulating strains in the region have to be assessed. This information is scarce in Tanzania.

Therefore the aims of the present study were to investigate a dairy herd experiencing abortion in order to:

- Establish within-herd prevalence of *Brucella* seropositive animals.
- Isolate, identify and characterize *Brucella* spp. from milk and abortion materials.
- Compare molecular characteristics of the obtained isolates with other strains in the region and outside.
- Investigate a possible spillover to small ruminants and dogs as they are a potential source of infection to cattle and can as well acquire infection from cattle.

## Methods

The present study is part of an extensive project on infectious cause of reproductive disorders in dairy cattle. During sampling on the present study farm, abortions were encountered and became available for the current investigation.

### Study farm

The farm is located in Mbarali district in Mbeya region in the southern highlands of Tanzania. At the time of sampling the farm had a total of 350 cattle which were crossbreeds of Friesian and Ayrshire with Boran and Zebu, 130 goats, 90 sheep and six dogs. The animals mingled with close interactions among them. All cattle and small ruminants grazed in controlled areas. The study herd had minimal contacts with pastoral herds and other dairy herds around, most of which took place during the dry season. There was no history of vaccination against brucellosis on the study farm.

### Animal material

Samples included serum, milk and one aborted fetus including fetal membranes collected in 2012–2013. Cattle were purposively selected to include only those above 6 months of age, while sheep and goats were randomly selected. Blood samples were collected from 200 cattle aged above 6 months, 50 goats, 35 sheep and six dogs. All female cattle above 6 months of age (*n* = 187) and all breeding bulls were included (*n* = 13). About 5 ml of whole blood was collected aseptically into plain vacutainer tubes. Blood samples were left at room temperature for about 12 h to allow serum separation. Serum was then pipetted into sterile tubes. Individual milk samples were collected from 63 cows, altogether from both Rose Bengal Test (RBT) positive and negative cows in sterile containers and properly sealed. Both serum and milk samples were transported to the laboratory on ice and stored at −20 °C until analysis. The aborted fetus and fetal membranes were examined on the farm. The fetus was examined externally for gross lesions and then aseptically dissected for examination of its internal organs. Examination revealed a relatively fresh fetus and its gestation stage was estimated to be 6 months.

Samples from all visceral organs (liver, lungs, kidneys, spleen, heart and brain) including foetal membranes were collected in a sterile plastic bag and were tight sealed and thereafter preserved at −20 °C for bacterial culture and isolation.

### Ethical statement

The protocol for field studies and collection of animal materials was approved by Njombe and Mbarali districts veterinary and agricultural authorities. Farmers were informed of the study and their verbal consent was sought before commencement of data collection.

### Serological examination

#### Rose Bengal test

All sera from cattle, goats, sheep and dogs were tested for presence of *Brucella* antibodies using RBT antigen following the manufacturer’s instructions (Standardized *B. abortus* Rose Bengal Test Antigen Central Veterinary laboratory New Haw Addlestone, Surrey, UK), in accordance with the OIE manual [1]. *Brucella* positive control serum was always included in the test.

### Indirect ELISA

Serum samples from cattle, sheep and goats were analyzed for the presence of *Brucella* spp*.* specific antibodies using indirect ELISA commercial kits following manufacturer’s instructions (SVANOVA^®^*Brucella*-Ab I-ELISA Svanova Biotech AB-Uppsala). To monitor interassay variations, *Brucella* positive control serum was always included.

### Milk ring test

Individual milk samples from RBT positive cows were tested on farm using Milk Ring Test (MRT) antigen (Atlas Medical William James House, Cambridge, UK) following the manufacturer’s instructions and in accordance with the OIE manual [1]. Due to shortage of reagents in the field, only ten milk samples were tested.

### Bacterial culture, isolation and identification

Bacteriological analysis was performed in a safety level-3 bio-containment facility at the Norwegian Veterinary Institute. Nineteen individual milk samples and aborted fetal organs as well as fetal membranes from one aborted fetus were subjected to bacterial culture. Primary isolation of *Brucella* spp. was done by inoculating the samples on a *Brucella* selective media (Selective Serum Dextrose Agar (SSDA)) (Oxoid) and Farrell’s medium. Two plates per sample, (one per medium) were used. From milk samples, 100 μL of milk were inoculated per plate. All plates were incubated both aerobically, and in 5 % CO_2_ atmosphere, at 37 °C and examined regularly after two, and up to 14 days, for *Brucella* like colonies. Such colonies were examined further with Gram staining. The plates were discarded if no growth was evident after 14 days of inoculation. Colonies typical of *Brucella* spp. were sub-cultured from which subsequent bacterial isolates were examined under phase contrast microscope and by Gram staining for organism morphology and size. Typical colonies revealing small Gram-negative coccobacilli, were further analyzed to obtain full identification and biotype.

### Classical biotyping

Classical biotyping was done as described by [21] at The National Reference Center for Brucellosis, Veterinary and Agrochemical Research Centre (CODA-CERVA) in Belgium. *Brucella* monospecific antisera A and M and *Brucella* phages Tb, Wb and Iz obtained from FAO/WHO Collaborating Center for Brucellosis Reference and Research at the Veterinary Laboratory Agency, Weybridge, UK were used. A panel of biotyping tests were performed and interpretation of the results was performed according to the OIE manual [1].

### DNA preparation and PCR

Suspected *Brucella* spp. isolates were subjected to genomic DNA extraction by heat treating a loopful of bacterial material dissolved in MQ water at 99 °C for 15 min [22]. After centrifugation, the supernatant was used as DNA template.

### Molecular identification

The extracted DNA was subjected to real time PCR for the *Brucella* spp. specific targeting IS*711* [13]. Primers and probe were developed at the Swedish Institute for Communicable Disease Control (unpublished protocol). Positive results were obtained for the three extracted DNA (results not shown).

### Bruce-ladder analysis

Species-level molecular identification was undertaken by multiplex PCR (Bruce-ladder) which was performed as described [23, 24] with the following conditions: Step 1: 95 °C 15 min, Step 2: 94 °C 30 s, Steps 3: 58 °C 90 s, Step 4: 72 °C 3 min, Step 5: 72 °C 10 min. Step 2, 3 and 4 was repeated in 25 cycles. The size of the PCR products was analyzed by capillary electrophoresis with Bioanalyzer^®^, Agilent Technologies, Santa Clara, CA, USA.

### MLVA-VNTR genotyping

The isolates identified as *B. abortus* biovar 3 were analyzed using MLVA-VNTR 16 loci as described before [14]. Primers used were those described by Le Fleche et al. [14]. A PCR master mix was prepared using the following reactives: buffer (10×), bethain, dNTP 2.5 mM, Taq DNA polymerase rec (5U/ul Invitrogen), MgCl2 and H_2_O. The following PCR program with iCycler BioRad was used: Step 1: 96 °C 5 min, Step 2: 96 °C 30 s, Step 3: 60 °C 30 s, Step 4: 70 °C 1 min, Step 5: 70 °C 5 min, Step 6: 8 °C. Step 2, 3 and 4 was repeated in 30 cycles. For the markers bruce 06, bruce 11, bruce 42, bruce 55 with repeat unit size 134 bp, 63 bp, 125 bp and 40 bp respectively, the PCR fragment size was analyzed by 2 % agarose gel electrophoresis. For the markers bruce 08, bruce 12, bruce 43, bruce 45, bruce 18, bruce 19, bruce 21, bruce 04, bruce 07, bruce 09, bruce16 and bruce 30, the size of the PCR products were analyzed by capillary electrophoresis with the CEQ 8000 Genetic Analysis System (Beckman Coulter, Indianapolis, IN, USA). The size of the PCR products were then converted to a corresponding tandem repeat number for each locus as described by Le Fleche et al. [14] to get the genotype.

To classify the Tanzanian *Brucella* strains, a polyphasic strategy that included phenotypic (classical biotyping) as well as genomic criteria (presence of IS*711*, Bruce-ladder and MLVA) was used. Accordingly, MLVA analysis within *B. abortus* biovar 3 was performed. The profile of the Tanzanian strains were compared to *B. abortus* biovar 3 genotypes deposited in the *Brucella* aggregated database on MLVAnet (http://mlva.u-psud.fr/) hosted by the Université Paris-Sud. Four *B. abortus* biovar 3 genotypes from Belgian strains were also included in the analysis.

Cluster analysis of MLVA data was performed with the software BioNumerics 2.1 (Applied Maths, Sint-Martens-Latem, Belgium) following previous methods by Le Fleche et al. [14]. Cluster analysis was done with Euclidean distance which gives the quantitative difference. Only isolates of 100 % similarity with the same number of tandem repeats in each locus were assigned to the same cluster. The most similar strains clustered closely together with short and thick edges, while the strains with high genomic variations had thin and longer edges. The dendrogram was generated using a distance matrix calculated with the categorical coefficient and the unweighted-pair group method using average linkages as previously described [14]. An identical weight was given to each marker. The MLVA profile of the isolates was also subjected to a minimum spanning tree (MST) analysis in BioNumerics (MLVA plugin 2.1), illustrating the relationship and possible mutation pathways within the clusters based on single locus variations (SLV). Only the units (and not the sizes) from each marker were considered for the analysis. The nodes (circles) consist of identical genotypes and the edges (lines) of weight based on number of mutations (steps) taken from the loci were used. Long weight (steps) indicates multiple mutations while short weight indicates few mutations.

## Results

### Serological findings

Ninety six out of 200 serum samples from cattle were positive in ELISA giving a within-herd prevalence of 48 % (95 % CI 41–55), while 43 of the 200 serum samples were positive with RBT resulting into within-herd prevalence of 21.5 % (95 % CI 16–27). Thirty six sera were positive in both tests, 60 were positive in ELISA but negative in RBT, seven were negative in ELISA but positive in RBT. All 10 milk samples were positive in MRT. All sheep goat and dog sera were negative in RBT. However, two out of 35 sheep (prevalence: 5.7 %; 95 % CI 0–17) and one out of 50 goat (prevalence: 2 %; 95 % CI 0–7) sera were positive in the ELISA.

### Bacterial culture, isolation, identification and biotyping characteristics

Three isolates of *Brucella* spp. were obtained, one from the aborted fetal liver and two from fetal membranes (all from the same animal). No *Brucella* spp. was isolated from milk samples. Real time PCR (IS*711*) confirmed the three isolates as *Brucella* spp. Bruce-ladder identified the isolates as *B. abortus* wild type as five fragments of 152, 450, 587, 774 and 1682 bp in sizes were amplified. The isolates showed common phenotypic characteristics typical for the genus *Brucella*. They grew anaerobically, in a 5 % CO_2_ atmosphere, and aerobically after 3–14 days incubation at 37 °C. Bacterial colonies were small, convex, and regular with smooth surface, honey colored, shiny and translucent. The organisms were gram negative, small (0.5–1 μm wide) single coccobacilli. The isolates were catalase and oxidase positive, also producing urease but not before 24 h of incubation. They were all H_2_S positive, and were agglutination with the monospecific anti-A serum but not the monospecific anti-M serum. The isolates grew in the presence of Thionine, Fuchsin and Safranin dyes. They were lysed by Tb both in RTD and RTD10^4^, Wb and Iz phages. The Tanzanian strains were characterized as *B. abortus* biovar 3, although they did not require CO_2_ for growth.

### MLVA genotyping

**Fig. 1:**
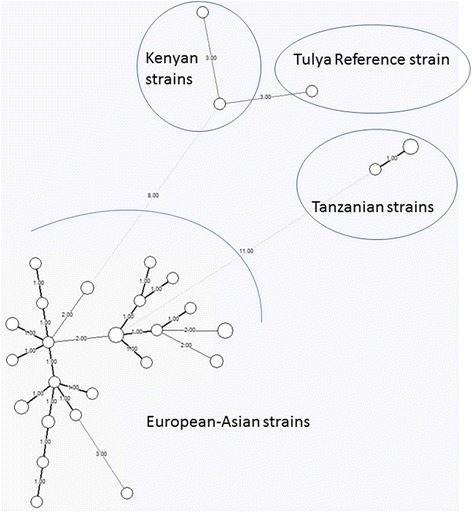
Minimum Spanning Tree (MST) analysis of the MLVA-16 profiles of three *Brucella abortus* biovar three genotypes isolated from cattle in Tanzania, compared with *Brucella abortus* biovar three strains isolated worldwide. MLVA profiles were determined for a selection of 28 *B. abortus* biovar 3 genotypes including the Tulya reference strain (MLVA profiles derived from publicly available data (http://mlva.u-psud.fr/), four recent Belgian isolates) and the three Tanzanian isolates. Clustering by minimum spanning tree was performed with Bionumerics. Circles outline the genetic profiles of strains. Numbers on the connecting lines refer to the number of markers differing between samples. The size of the circles is proportional to the number of strains (1 or 2) bearing the same genetic profile

**Fig. 2:**
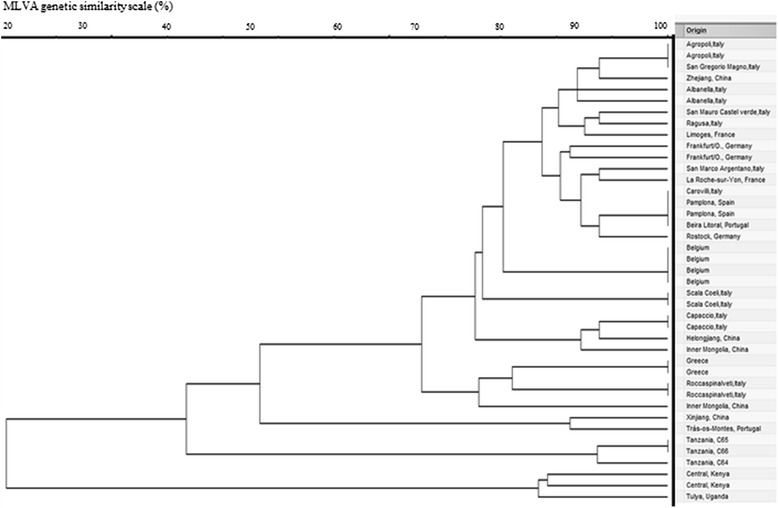
Clustering analysis of 40 *B. abortus* field strains and Tulya *B. abortus* biovar 3 reference strain with the two panels of markers (MLVA-16). Scale (%) shows the MLVA genetic similarity. The geographic origin is given in the column. All strains were isolated from cattle, except one of the Belgian strain that was isolated from a dog in the farm where *B. abortus* biovar 3 was isolated from cattle and the Tulya reference strain originally isolated from a human patient in Uganda in 1958

The MLVA 16 loci identified three closely related *B. abortus* biovar 3 Tanzanian genotypes (C64, C65 and C66). Out of the three isolates, C65 and C66 were identical while C64 was different at one locus (Table 1). The three isolates were identical using panel 1 loci but different at the locus Bruce 16 in panel 2. There was no amplification at locus Bruce 19 for strain C64. The genotypes were different from the reference strain’s genotype and from genotypes from Kenya. Despite the Tanzanian genotypes being unique, they were more closely related to genotypes originating from Europe, Turkey and China than to genotypes from Uganda and Kenya (Figs. 1 and 2).

**Table 1:** Sixteen loci variable number of tandem repeat for the three Tanzanian *Brucella abortus* genotypes (C64, C65 and C66)

		*B. abortus* biovar 3			
	Locus	C64	C65	C66	Reference Tulya
Panel 1	Bruce 06	2	2	2	3
	Bruce 08	4	4	4	5
	Bruce 11	2	2	2	4
	Bruce 12	12	12	12	11
	Bruce 42	3	3	3	2
	Bruce 43	2	2	2	2
	Bruce 45	3	3	3	3
	Bruce 55	3	3	3	3
	Bruce 19	^a^	42–44	42–44	40
Panel 2	Bruce 04	7–8	7–8	7–8	6
	Bruce 07	2	2	2	5
	Bruce 09	6	6	6	3
	Bruce 16	7	8	8	11
	Bruce 18	5	5	5	8
	Bruce 21	8	8	8	8
	Bruce 30	4	4	4	5

*B. abortus* biovar 3 strain Tulya was used as reference strain

^a^indicates no amplification

## Discussion

High within-herd seroprevalence in affected cattle herds has also been reported in Nigeria and Uganda [20, 25]. In Uganda within-herd prevalence of seropositive animals varied from 1 to 90 % [26]. Management systems such as common grazing increase the contact between cattle. Extensive movement and sharing of pasture enhances contact of cattle from different areas and facilitates transmission of most infectious diseases, including brucellosis. However, other studies have shown that management systems, where cattle are in constant movement puts a natural limit on the rate of *Brucella* infection accumulation or transmission and that the prevalence decreases [27, 28].

Positive MRT for individual animals in the herd suggests infection, but mastitis and colostrum might have caused false positivity [1] as 65 % of animals from this herd gave positive results in a California mastitis test (results not shown).

Only few of the small ruminants had antibodies against *Brucella* spp. The presence of seropositive small ruminants from mixed farming systems has been reported by others in Tanzania [19], Uganda [29] and Ethiopia [30], while in Togo, no seropositive small ruminants in mixed farming systems with seropositive cattle were found [31]. The presence of *B. abortus* in cattle, high within-herd seroprevalence, and the small number of seropositive small ruminants suggests a spillover of *B. abortus* from cattle to small ruminants, although the presence of *B. melitensis* or its DNA was not investigated in the later species [32]. In case of *B. melitensis* infection in small ruminants, given the *B. melitensis* basic reproductive number (R_0_) of 1.2 (if greater than 1, the number of infected animals increases) as calculated in Mongolia [28], a higher seroprevalence should have been seen in small ruminants in our study. Such an epidemiological situation has recently been described in the Sudan were *B. abortus* biovar 6 had spilled over from cattle to sheep [32]. Naturally acquired *B. abortus* infection in dogs associated with infected cattle has been reported [33]. Although dogs may be valuable indicators (sentinels) of brucellosis in cattle, this study suggests that dogs did not play any significant role in the epidemiology of bovine brucellosis in this farm.

Both RBT and ELISA are OIE prescribed screening tests for brucellosis [1]. In the present study, both tests were used in parallel in cattle. Discrepancies between the two tests in the present study could be due to several reasons, including differences in sensitivity and specificity as indirect ELISA (iELISA) was reported to be more sensitive than RBT [5] and RBT more specific than the iELISA [34]. In some studies, iELISA has been shown to detect more cattle chronically infected with *Brucella* than the RBT [9]. It is worth noting that cut-off points for iELISA have been defined for use in *Brucella*-free regions to optimize sensitivity [34]. The performance of such tests in endemic regions such as sub-Saharan Africa is unknown. Commercial ELISA kits need thus to be evaluated and cut-offs need to be established for specific epidemiological regions. The present iELISA results may therefore overestimate the actual within-herd seroprevalence. With this word of caution, the results suggest that there is a high within-herd prevalence and indicate that cattle in this farm were chronically infected.

This is the first report on the isolation, identification and characterization of *B. abortus* biovar 3 from cattle in Tanzania. It is important to isolate and characterize *Brucella*, as with serological methods it is not possible to infer which smooth *Brucella* spp. induced antibodies in the host [6, 9]. Some serological tests lack sensitivity and it is impossible to differentiate antibodies produced after vaccination from those produced after infection [5].

Biotyping profiles of the isolated strains indicated characteristics typical for *B. abortus* biovar 3, except CO_2_ requirement for growth [21]. However *B. abortus* biovar 3 reference strain Tulya, which was isolated from a human patient in the neighboring country Uganda, is reported to be CO_2_ independent [35]. Growth in the absence of CO_2_ has been observed to occur within the same biovars [36, 37].

In the present study, two different genotypes were obtained from the same animal. To the best of the author’s knowledge, both genotypes have never previously been described. The genetic polymorphism observed is incongruent with that observed in Uganda [38] and Kenya [7]. The genetic polymorphism shown at the panel two at one locus that is usually polymorphic might explain the difference between the two genotypes. Both genotypes are more related to European and Asian genotypes than to African genotypes. This suggests that the Tanzanian genotypes were introduced from Europe, possibly through importation of infected animals, although the time frame when this occurred remains elusive.

*Brucella abortus* biovar 3 has also been isolated from other African countries including Kenya, Gambia and recently Togo [4, 7, 36]. In Zimbabwe and Nigeria, *B. abortus* biovar 1 and 2 are common while *B. abortus* biovar 3 is rare [3, 37]. Specific biovars of *Brucella* are said to predominate certain geographical regions with *B. abortus* biovar 3 being commonly encountered in cattle in Africa [39]. In Egypt, infection of cattle by *B. melitensis* [40] and recently also *B. abortus*, and *B. suis* [15] have been reported. Some authors have proposed using AMOS-ery PCR to divide biovar 3 into two groups: one group 3a that will contain strain Tulya and field strains isolated from Africa while group 3b will contain strains from Europe [13]. This was not performed in the present study but the MLVA results suggest that the Tanzanian strains are not related to strain Tulya. Hence classifying *B. abortus* biovar 3 strains according to their geographical origin should be carefully considered.

The absence of culture positive milk samples could be due to the low number of samples tested, too few bacteria in the sample, or due to a low volume of milk inoculated. The excretion of organisms in milk is intermittent [21]. Freezing of milk might also have been a negative factor since the bacteria are easier to culture from fresh samples or samples stored at refrigeration temperature [41]. Consumption of raw milk is practiced in some communities in Tanzania. Under such conditions brucellosis is a public health issue.

This report further highlights the role of *Brucella* spp. as cause of reproductive problems on this farm, as the bacteria were cultured from the aborted fetus and associated membranes. In addition, large and medium scale dairy farms represents a risk for spread of the bacteria to other herds as they are sources of replacement heifers to small-scale dairy herds.

## Conclusion

This is the first isolation, identification and characterization of *B. abortus* biovar 3 from a cow in a dairy herd in Tanzania. In the absence of any control program, the isolation of the pathogen and the high within-herd prevalence suggest chronic infection in this herd. Importantly, big herds like this serves as potential sources of replacement heifers to smallholder farmers, posing risk of infection transmission to other herds. Since *B. abortus* is a zoonotic agent, there is a risk of transmission to humans hence further studies on human brucellosis in the region are recommended. Information on the prevalence and the circulating *Brucella* strains in different livestock species and possibly wildlife is important to understanding transmission patterns and risk factors. It is also a necessary first step in designing appropriate control policies and strategies. The results suggest that the Tanzanian strains are not related to other *B. abortus* biovar 3 strains isolated in the neighboring countries, Uganda and Kenya. This highlights that transmission patterns in the region are virtually unknown. In order to decipher such transmission patterns in the region, more strains should be isolated and characterized.

## Abbreviations

NMBU: Norwegian University of Life Sciences
PCR: Polymerase chain reaction
AMOS-ery: Abortus, Melitensis, Ovis, Suis (with ery primers)
MLVA-VNTR: Multiple locus variable number of tandem repeat
MST: Minimum spanning tree
SLV: Single locus variation
iELISA: indirect Enzyme Linked Immunosorbent Assay
RBT: Rose bengal test
MRT: Milk ring test
DNA: Deoxyribonucleic acid
RTD: Routine test diagnostic
MQ: Milli-Q
SSDA: Selective serum dextrose agar
IS*711*: Insertion sequence 711
Tb: Tbilisi
Wb: Weybridge
Iz: Izatnagar
Bp: Base pair
